# *Helicobacter pylori* infection is associated with reduced risk of Barrett’s esophagus: a meta-analysis and systematic review

**DOI:** 10.1186/s12876-021-02036-5

**Published:** 2021-12-07

**Authors:** Yan-Lin Du, Ru-Qiao Duan, Li-Ping Duan

**Affiliations:** Department of Gastroenterology, Peking University Third Hospital, No. 49 North Garden Rd., Haidian District, Beijing, 100191 China

**Keywords:** *Helicobacter pylori*, Barrett’s esophagus, Gastroesophageal reflux disease

## Abstract

**Background:**

*Helicobacter pylori* (*Hp*) is a class I carcinogen in gastric carcinogenesis, but its role in Barrett’s esophagus (BE) is unknown. Therefore, we aimed to explore the possible relationship.

**Methods:**

We reviewed observational studies published in English until October 2019. Summary odds ratios (ORs) and 95% confidence intervals (CIs) were calculated for included studies.

**Results:**

46 studies from 1505 potential citations were eligible for inclusion. A significant inverse relationship with considerable heterogeneity was found between *Hp* (OR = 0.70; 95% CI, 0.51–0.96; *P* = 0.03) and BE, especially the CagA-positive *Hp* strain (OR = 0.28; 95% CI, 0.15–0.54; *P* = 0.0002). However, *Hp* infection prevalence was not significantly different between patients with BE and the gastroesophageal reflux disease (GERD) control (OR = 0.99; 95% CI, 0.82–1.19; *P* = 0.92). *Hp* was negatively correlated with long-segment BE (OR = 0.47; 95% CI, 0.25–0.90; *P* = 0.02) and associated with a reduced risk of dysplasia. However, *Hp* had no correlated with short-segment BE (OR = 1.11; 95% CI, 0.78–1.56; *P* = 0.57). In the present infected subgroup, *Hp* infection prevalence in BE was significantly lower than that in controls (OR = 0.69; 95% CI, 0.54–0.89; *P* = 0.005); however, this disappeared in the infection history subgroup (OR = 0.88; 95% CI, 0.43–1.78; *P* = 0.73).

**Conclusions:**

*Hp*, especially the CagA-positive *Hp* strain, and BE are inversely related with considerable heterogeneity, which is likely mediated by a decrease in GERD prevalence, although this is not observed in the absence of current *Hp* infection.

## Background

Owing to improvements in hygiene and living conditions, the prevalence of *Helicobacter pylori (Hp)* has continued to fall in developed countries, along with the incidence of gastric cancer and peptic ulcer, although it remains high in some developing countries, such as 70.1% in Africa [1, 2]. Interestingly, in contrast to the decline in the rate of *Hp* infection, the incidence of esophageal adenocarcinomas (EAC) has increased significantly. Current epidemiological studies present a consistent, rapidly increasing incidence of EAC in the United States and most other western countries, especially among males, with an observed or estimated start between 1960 and 1990, while the incidence of esophageal squamous cell carcinoma is stable or declining in all racial groups [3, 4]. The etiology of EAC is multifactorial, and Barrett’s esophagus (BE) is a premalignant lesion that is observed in the majority of patients with EAC, and carries a risk of eventual development of EAC that is up to 30- to 125-fold higher than that in patients without this condition [5, 6]. Previous studies have identified several risk factors for the development of BE, including male sex, older age, smoking, white race, obesity, hiatal hernia, and gastroesophageal reflux disease (GERD) [7, 8]. However, the possible role of *Hp* in BE is uncertain. Currently, *Hp* is classified by the World Health Organization as a class 1 carcinogen, since it promotes gastric cancer, and is also regarded as a commensal organism that confers some protection against asthma, allergies, and even obesity [9, 10]. *Hp* seems to have a protective influence on BE, however, the relationship between *Hp* and BE remains controversial.

Multiple studies have highlighted the relationship between *Hp* and BE [11–13]. Recently, Wang used individual-level data from six case–control studies to conduct analysis. Their study provided evidence that *Hp* infection was strongly inversely associated with BE, which was even stronger among individuals with cytotoxin-associated gene A (CagA) positive strain [14]. Another extensive meta-analysis also demonstrated that *Hp* infection was associated with a reduced risk of BE, and dysplastic, non-dysplastic, and long-segment BE (LSBE), and demonstrated that the risk reduction was not correlated with geographical location [15]. However, some researchers concluded that there was no clear association between *Hp* and BE, or demonstrated contrary conclusions in case–control studies and cohort studies [16, 17]. Fischbach’s meta-analysis of 49 observational studies identified a protective effect of *Hp* on BE, and showed great heterogeneity between the majority of studies, which was potentially due to selection and information bias [18]. Consequently, it is understandable that different meta-analyses come to different conclusions.

Previous meta-analysis results are inconsistent, and the heterogeneity between them may derive from selection of the control group, the definition of BE, and the *Hp* detection method. To better understand this relationship, we performed meta-analysis and subgroup analysis based on the potential sources of heterogeneity. This study would contribute to the design of clinical studies and the decisions on whether to eradicate *Hp*.

## Methods

### Search strategy

PubMed, EMBASE, and COCHRANE databases were searched from inception to October 2019. We used the following MeSH terms or keywords as search terms: (("Barrett Esophagus"[Mesh]) OR (Barrett metaplasia) OR (Barrett metaplasias) OR (Barrett’s Metaplasia) OR (Metaplasia, Barrett) OR (Metaplasias, Barrett) OR (Barrett’s Syndrome) OR (Barretts syndrome) OR (Barrett Syndrome) OR (Barrett’s Esophagus) OR (Barrett’s oesophagus) OR (Barretts Esophagus) OR (Barretts oesophagus) OR (Esophagus, Barrett’s) OR (oesophagus, Barrett’s) OR (Esophagus, Barrett) OR (oesophagus, Barrett) OR (Barrett Epithelium) OR (Epithelium, Barrett) OR (Barrett’s) OR (Barrett)) AND (("*Helicobacter pylori"*[Mesh]) OR (*Helicobacter pylori*) OR (*H pylori*) OR (*H. pylori*) OR (*Helicobacter*) OR (*Campylobacter*)) AND (Humans).

### Inclusion and exclusion criteria

All eligible studies satisfied the following inclusion criteria:

1. Observational studies: Case–control, cohort, or cross-sectional studies
2. Providing raw data on *Hp* infection in the BE and control groups
3. Studies conducted in adult populations

Studies with the following exclusion criteria were eliminated:

1. Full-text articles in languages other than English
2. Studies in which the data came from a review article or other non-full-text article
3. Less than five points in the Newcastle–Ottawa Scale (NOS)

When the same data appeared in different articles, only the study with the most complete relevant data was included.

### Data extraction

Data were extracted by two independent investigators after reading each included study. When agreement was reached by discussion or with the help of third investigators, the data were recorded in a designed Excel 2019 sheet. We collected data on author, year of publication, journal, geographical location, study type, *Hp* infection testing methods, definition of cases and controls, number of cases and controls, number of *Hp* infections in cases and controls, and whether matched in sex, age, obesity, smoking, alcohol, and race. Data on dysplasia, segment length and infection of CagA-positive *Hp* strain were included when present. When the subjects of multiple reports are the same. Only one, the most complete, would be included.

### Statistical analysis

Our primary objective was to compare the prevalence of *Hp* infection between BE groups and controls. The secondary objective was to conduct subgroup analysis according to the differences in definitions of the control group, the definitions of BE, and the *Hp* detection methods, in order to clarify the impact of these aspects on the overall results. The correlation between *Hp* and BE was determined by calculating the odds ratios (ORs) and 95% confidence intervals (CIs) for risk. The results of the meta-analysis were displayed on a forest map, heterogeneity was assessed using Cochrane's Q and I^2^ statistics, and publication biases were checked by visual assessment of funnel plots. Heterogeneity was regarded as moderate, substantial, and considerable when the I^2^ was between 30–60%, 50–90%, and 75–100%, respectively. All calculations were conducted by Review Manager 5.3.

## Results

**Fig. 1:**
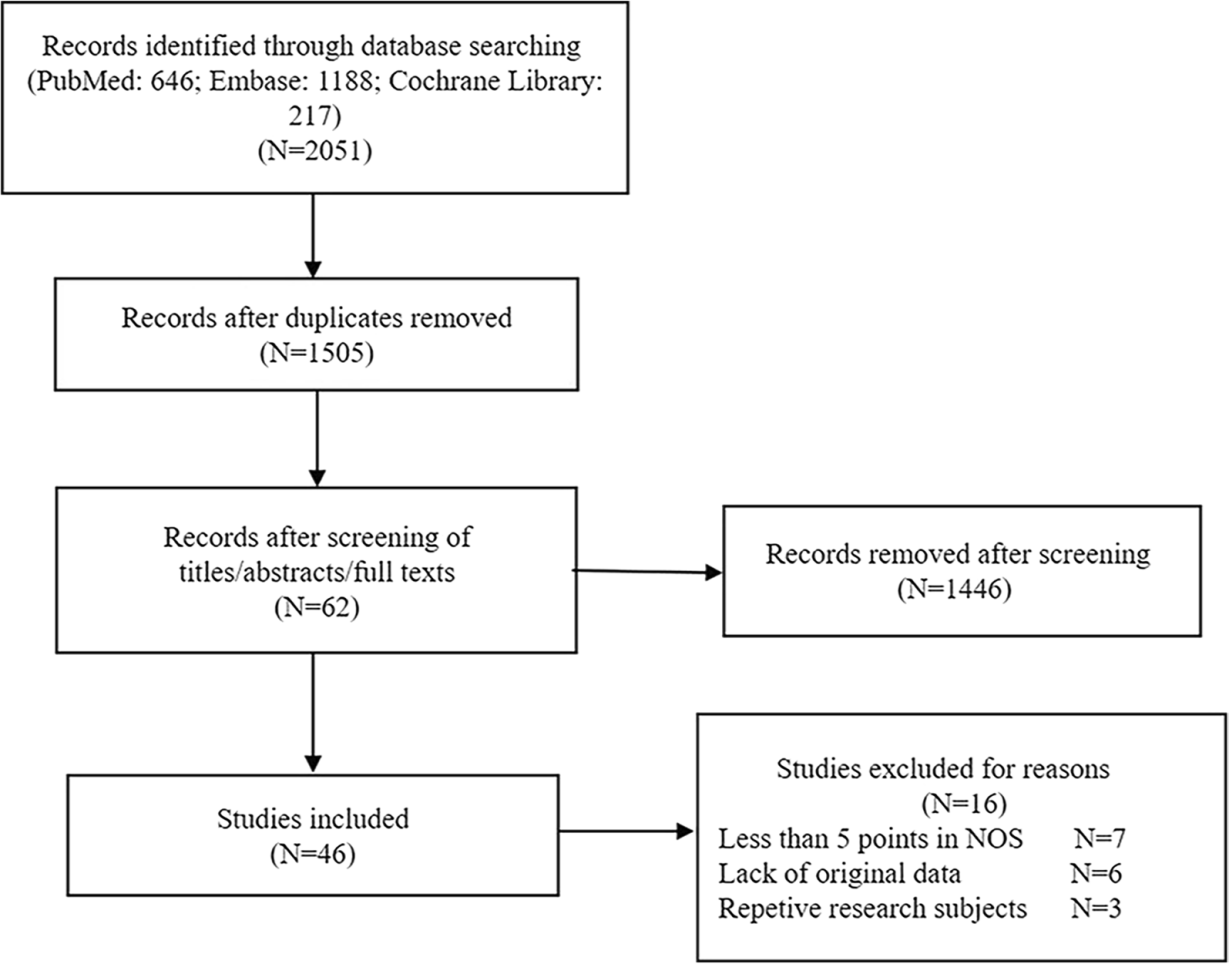
Flow chart of the study selection process

Searches initially generated 1505 potential citations after removing 546 duplicates from 2051 citations. A large sample study (n = 1445) was further excluded by screening titles, abstracts, and browsing full-text. A total of 62 studies remained for full-text review, and six studies without original data [19–24]. and seven studies with less than five points in NOS were additionally excluded [25–31]. Three studies were excluded because of repetitive research subjects [32–34]. Finally, Forty-five studies were included in this article; data from 36 of these were extracted to explore the relationship between *Hp* and BE, while others examined the correlation in *Hp* and BE dysplasia, lengths of BE, and the correlation between the CagA-positive *Hp* strain and BE. The study selection process is shown in Fig. 1.

### Prevalence of *Hp* infection in BE and controls

**Fig. 2:**
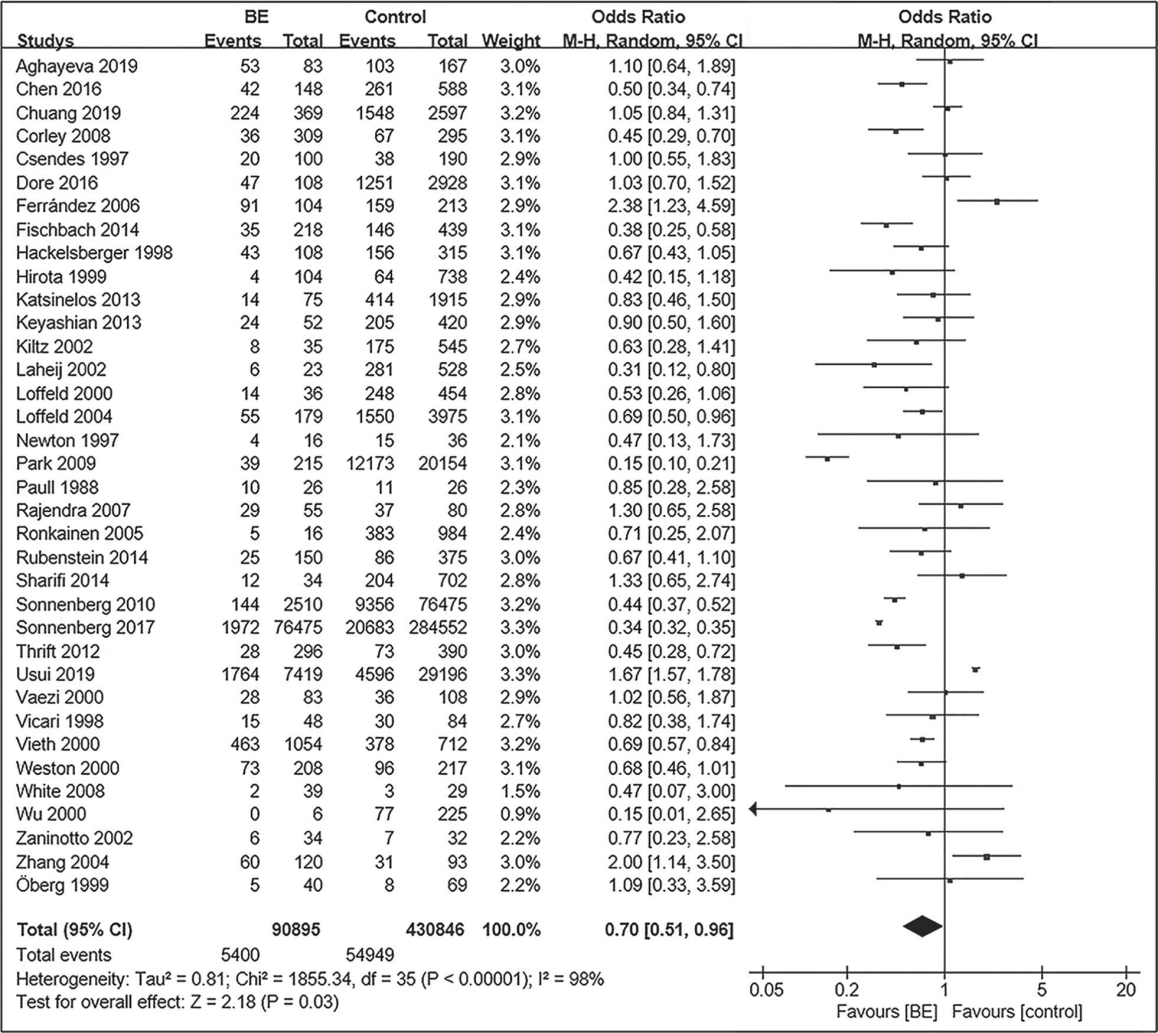
Forest plot of the random effect analysis of the 36 studies. The weights and heterogeneities of studies are indicated too. OR: Odds ratio, CI: 95% confidence interval

**Fig. 3:**
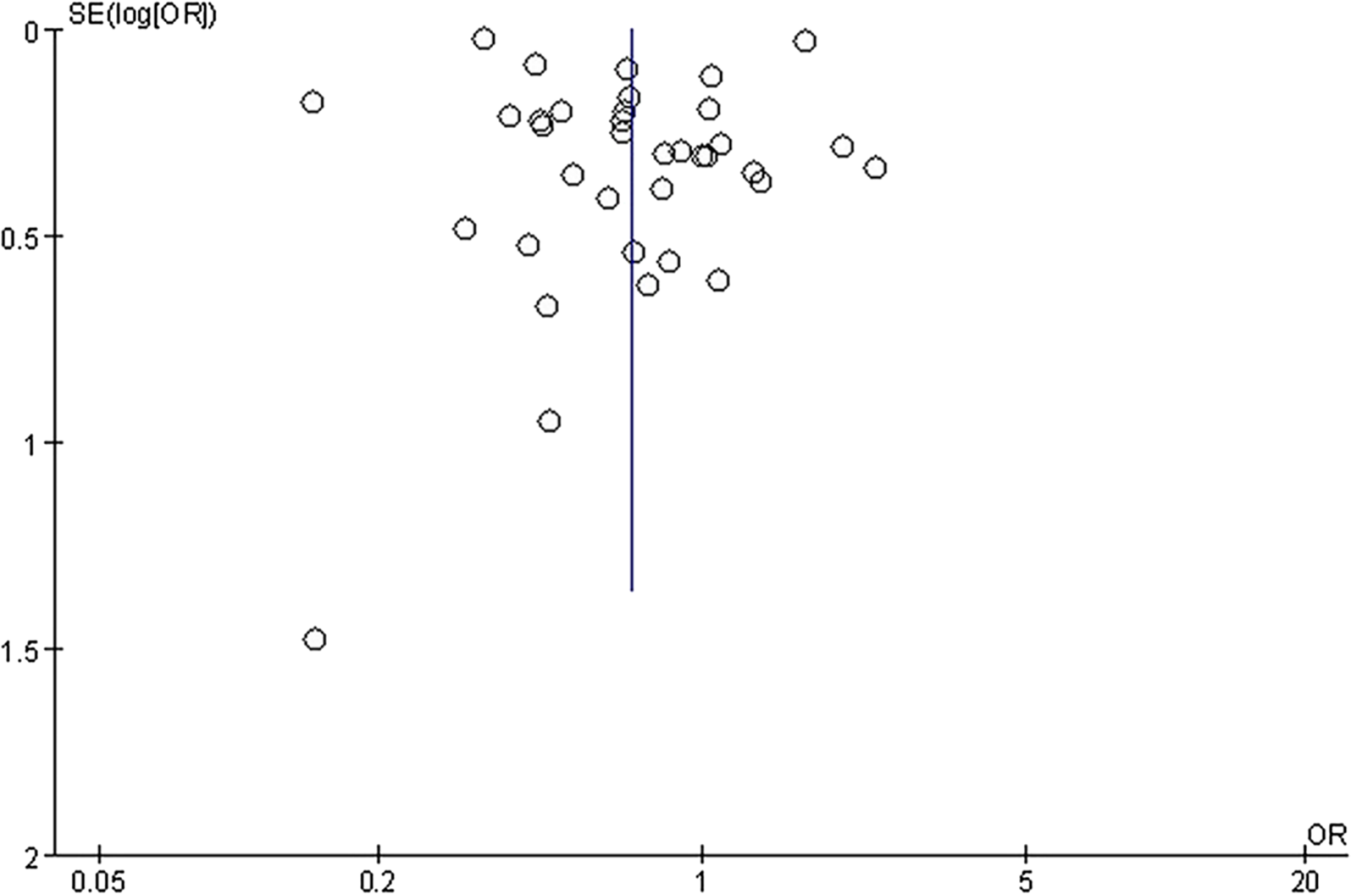
Funnel plot of the random effect analysis of the 36 studies

**Fig. 4:**
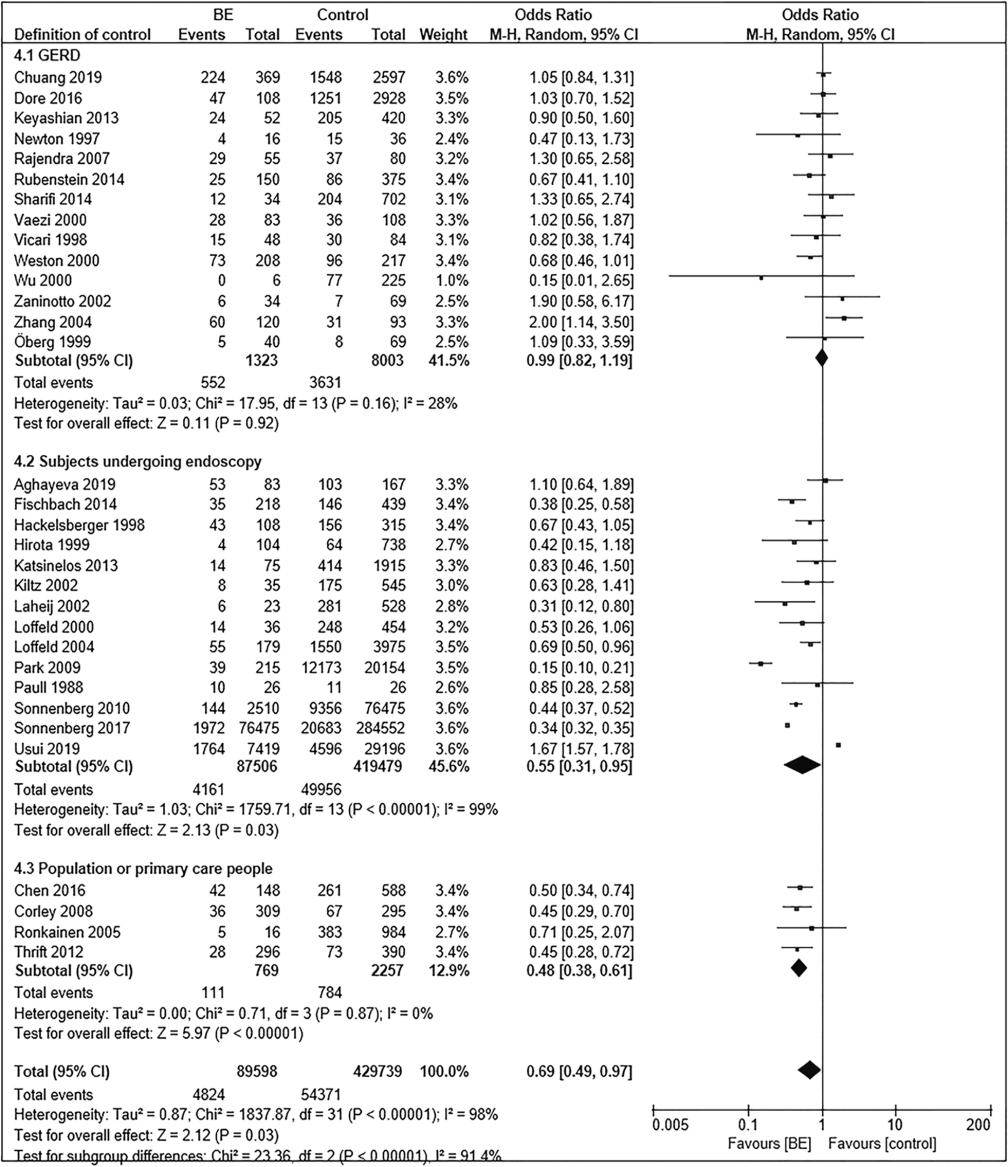
Forest plot of subgroup analysis according to definition of control group

**Fig. 5:**
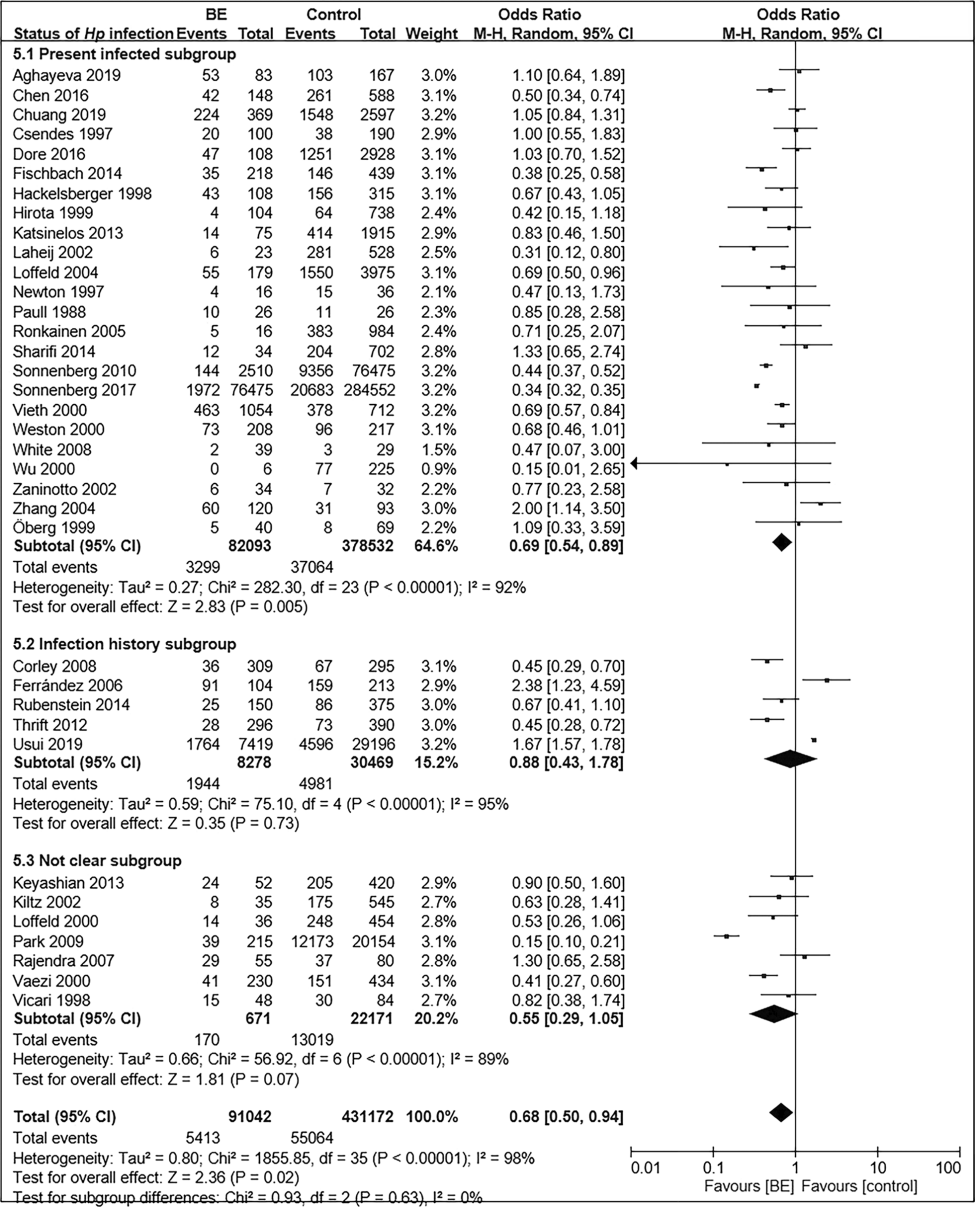
Forest plot of subgroup analysis according to status of *Hp* infection. 5.1: *Hp* positive with rapid urease test, urea breath test, histology or culture; 5.2: *Hp* positive with serological detection, treatment history, or infection history; 5.3: not sure to status of *Hp* infection

The 36 included studies comprised a total of 90,895 BE patients and 430,846 controls [11–13, 35–67]. A summary of the characteristics of these studies is shown in Table 1. The prevalence of *Hp* infection in BE patients was significantly lower than that in controls (OR = 0.70; 95% CI, 0.51–0.96; *P* = 0.03), with considerable heterogeneity observed between studies (I^2^ = 98%, *P* < 0.00001) (Fig. 2). Funnel plots suggested no obvious publication bias (Fig. 3). Subgroup analysis was conducted according to differences in definition of control group. Fourteen studies regarded patients with GERD as control group [37, 43, 49, 52, 54, 55, 58–60,62,63, 64,66, 67]. There was no significant difference in the prevalence of *Hp* infection between BE and GERD controls (OR = 0.99; 95% CI, 0.82–1.20; *P* = 0.91; I^2^ = 33%). In contrast, the negative relationship between *Hp* prevalence and BE was enhanced when defining subjects undergoing endoscopy in another 14 studies (OR = 0.55; 95% CI, 0.31–0.95; *P* = 0.03; I^2^ = 99%) or normal control (population or primary care people) in four studies (OR = 0.48; 95% CI, 0.38–0.61; *P* < 0.00001; I^2^ = 0%) as control groups (Fig. 4) [11, 13, 35, 36, 38, 40–42, 44–48, 50, 51, 53, 56, 57]. When BE was defined as intestinal metaplasia (IM) in 26 studies, we found an increased negative correlation between *Hp* prevalence and BE (OR = 0.64; 95% CI, 0.51–0.80; *P* = 0.0001; I^2^ = 90%) [11, 12, 13, 36, 38, 40, 42–45, 50, 52–58, 60–67]. However, the negative correlation disappeared (OR = 0.76; 95% CI, 0.51–1.14; *P* = 0.18; I^2^ = 92%) in the other subgroups, which diagnosed BE with columnar metaplasia (CM), endoscopic presentation, no clear definition, and gastric epithelium [35, 37, 39, 41, 46–49, 51, 59]. In addition, we divided the studies according to whether *Hp* could be confirmed as a present infection, into the present infected subgroup (*Hp* positive with rapid urease test, urea breath test, histology, or culture), infection history subgroup (*Hp* positive with serological detection, treatment history, or infection history), and not clear subgroup. In the present infected group with 24 studies, the prevalence of *Hp* infection in BE was significantly lower than that in controls (OR = 0.69; 95% CI, 0.54–0.89; *P* = 0.005; I^2^ = 92%) [11, 13, 36, 37, 39–44, 46,48, 49, 51, 53, 55, 56, 60–63, 65–67], while the negative correlation disappeared again in the infection history subgroup (OR = 0.88; 95% CI, 0.43–1.78; *P* = 0.73; I^2^ = 95%) (Fig. 5) [12, 35, 38, 54, 57].

**Table 1:** Characte ristics of the 36 studies included ro research the correlation between *Hp* and BE

Authors	Years	Journal	*Hp * testing meth od	Biopsy locati on	BE	Control	Sex match	Age m atch	BMI/obesity match	Smoking match	Alcohol match	Race match
Aghayeva et al. [36]	2019	Dis Esophagus	H^*^, R^†^	Antrum	IM^‡^	Endoscopy	Yes	Yes	Not clear	Not clear	Not clear	Yes
Chen et al. [13]	2016	PLoS One	R	Antrum	IM	Primary care	Yes	Yes	Not clear	Not clear	Not clear	Not clear
Chuang et al. [37]	2019	Kaohsiung Journal of Medical Sciences	H, R, U^§^	Not clear	Not clear	GERD^||^	Not clear	Not clear	Not clear	Not clear	Not clear	Not clear
Corley et al. [38]	2008	Gut	S^¶^		IM	Population	Yes	Yes	Not clear	Not clear	Not clear	Not clear
Csendes et al. [39]	1997	Dis Esophagus	H	Antrum	Gastric epithelium ≥ 3 cm or IM	Endoscopy, Primary care	No	No	Not clear	Not clear	Not clear	Not clear
Dore et al. [63]	2016	Scand J Gastroenterol	H, R, 13C-UBT	Antrum, Angulus, Corpus	IM	GERD	Not clear	Not clear	Not clear	Not clear	Not clear	Not clear
Ferrández et al. [12]	2006	BMC Gastroenterol	S		IM	Blood donor	Yes	Yes	Not clear	No	No	Not clear
Fischbach et al. [40]	2014	Am J Gastroenterol	H, C^**^	Antrum, Corpus, Cardia	IM	Endoscopy	Yes	Yes	Yes	No	Not clear	No
Hackelsberger et al. [41]	1998	Gut	H, R	Antrum, Corpus	Endoscopic diagnose	Endoscopy	Not clear	Not clear	Not clear	Not clear	Not clear	Not clear
Hirota et al. [42]	1999	Gastroenterology	H	EGJ^††^	IM	Endoscopy	Not clear	Not clear	Not clear	Not clear	Not clear	Not clear
Katsinelos et al. [44]	2013	Hippokratia	R	Antrum	IM	Endoscopy	Yes	Yes	Yes	Yes	Yes	Not clear
Keyashian et al. [64]	2013	Dis Esophagus	H, S, stool antigen	Not clear	IM	GERD	No	No	Yes	Yes	Not clear	Not clear
Kiltz et al. [45]	2002	Eur J Gastroenterol Hepatol	R, S	Antrum, Corpus	IM	Endoscopy	Not clear	Not clear	Not clear	Not clear	Not clear	Not clear
Laheij et al. [46]	2002	Alimentary Pharmacology and Therapeutics	H, R, C	Antrum	CM^‡‡^	Endoscopy	No	Not clear	Not clear	Not clear	Not clear	Not clear
Loffeld et al. [47]	2000	Digestion	H, R, S, C	Antrum	CM	Endoscopy	Not clear	Not clear	Not clear	Not clear	Not clear	Not clear
Loffeld et al. [48]	2004	Netherlands Journal of Medicine	H, C	Antrum	Not clear	Endoscopy	Not clear	No	Not clear	Not clear	Not clear	Not clear
Newton et al. [49]	1997	Gut	R	A ntrum	Not clear	GERD	No	No	Not clear	Not clear	Not clear	Not clear
Öberg et al. [43]	1999	Archives of Surgery	H	Antrum, biopsies just below SCJ^§§^	IM	GERD	Not clear	Not clear	Not clear	Not clear	Not clear	Not clear
Park et al. [50]	2009	J Clin Gastroenterol	H, R, S	Not clear	IM	Endoscopy	No	No	No	No	No	Yes
Paull and Yardley [51]	1988	Gastroenterology	H	Gastric biopsy	Not clear	Endoscopy	Yes	Yes	Not clear	Not clear	Not clear	Not clear
Rajendra et al. [52]	2007	Helicobacter	H, R, S	Antrum, Corpus, Cardia	IM	GERD	Not clear	Not clear	Not clear	Not clear	Not clear	Not clear
Ronkainen et al. [53]	2005	Gastroenterology	H, C	Antrum, Corpus	IM	Population	Not clear	Not clear	Not clear	No	No	Not clear
Rubenstein et al. [54]	2014	Clin Gastroenterol Hepatol	S		IM	GERD	Yes	Not clear	Not clear	Not clear	Not clear	Not clear
Sharifi et al. [55]	2014	Gastroenterol Res Pract	R	Antrum	IM	GERD	Yes	No	No	Yes	Yes	Not clear
Sonnenberg et al. [56]	2010	Gastroenterology	H	Stomach	IM	Endoscopy	No	No	Not clear	Not clear	Not clear	Not clear
Sonnenberg et al. [11]	2017	Aliment Pharmacol Ther	H	Stomach	IM	Endoscopy	No	No	Not clear	Not clear	Not clear	No
Thrift et al. [57]	2012	Int J Cancer	S		IM	Population	Not clear	Not clear	Not clear	Not clear	Not clear	Not clear
Usui et al. [35]	2019	J Clin Gastroenterol	S		Endoscopic diagnose	Endoscopy	Not clear	Not clear	Not clear	Not clear	Not clear	Not clear
Vaezi et al. [58]	2000	Am J Gastroenterol	H ,S	Antrum, Corpus	IM	GERD	Not clear	Yes	Not clear	Not clear	Not clear	Not clear
Vicari et al. [59]	1998	Gastroenterology	H, S	Antrum, Fundus, Cardia	CM ≥ 3 cm or IM	GERD	Not clear	Yes	Not clear	Not clear	Not clear	Yes
Vieth et al. [65]	2000	Digestion	H	Antrum, Corpus	IM	NUD^||||^	No	No	Not clear	Not clear	Not clear	Not clear
Weston et al. [60]	2000	Am J Gastroenterol	H	Stomach	IM	GERD	Yes	Yes	Not clear	Yes	Yes	No
White et al. [61]	2008	Can J Gastroenterol	H	Not clear	IM	Normal SCJ	No	Yes	Not clear	Not clear	Not clear	Not clear
Wu et al. [66]	2000	Alimentary Pharmacology and Therapeutics	H, R	Antrum, Corpus	IM	GERD	Not clear	Not clear	Not clear	Not clear	Not clear	Not clear
Zaninotto et al. [67]	2002	Dig Liver Dis	H	Esophagus	IM	GERD	No	No	Not clear	Not clear	Not clear	Not clear
Zhang et al. [62]	2004	World J Gastroenterol	H	Antrum	IM	GERD	Not clear	Not clear	Not clear	Not clear	Not clear	Not clear

*: Histology, †: Rapid urease test, ‡: Intestinal metaplasia, §: Urea breath test, ||: Gastroesophageal reflux disease, ¶: Serology, **: Culture, ††: Esophagogastric junction, ‡ ‡: Columnar metaplasia, §§: Squamous Columnar Junction, ||||: Non-ulcer dyspepsia

### Correlation between *Hp* and length of BE

We extracted data from 11 studies to explore the correlation between *Hp* and LSBE, and obtained a total of 669 BE patients and 31,243 controls [35, 42, 45, 58, 62, 67, 68–72]. We found that the risk of *Hp* infection in patients with LSBE was significantly lower than that in the controls (OR = 0.47; 95% CI, 0.25–0.90; *P* = 0.02; I^2^ = 82%). In contrast, we extracted data from 12 studies to explore the correlation between *Hp* and short-segment BE (SSBE), and obtained a t otal of 7886 BE patients and 31,173 controls [35, 36, 42, 45, 58, 62, 67, 73, 70, 74–76]. There was no significant difference in the prevalence of *Hp* between the SSBE and controls (OR = 1.11; 95% CI, 0.78–1.56; *P* = 0.57; I^2^ = 68%). Although the same *Hp* infection rate was observed in the ultra-short-segment BE (USBE) and GERD groups (22%, 2/9 vs. 22% 7/32) in Zaninotto’s study, such a small sample size might lead to bias [67]. Matsuzaki’s research suggested that the *Hp* infection rate in USBE was lower than that in controls, but the difference was not significant (66.3%, 57/86 vs 72.5%,50/69; *P* > 0.05) [76].

### Correlation between *Hp* and BE dysplasia

Only four previous studies have focused on whether *Hp* reduces the risk of BE dysplasia [11, 36, 57,65]. Decades ago, Vieth found that patients with BE neoplasia (high-grade dysplasia or EAC) had significantly lower rates of *Hp* infection than patients with non-ulcer dyspepsia (*P* < 0.01), which was also lower than that observed in patients with simple BE [65]. This conclusion was further confirmed by two subsequent studies. In a population-based case–control study, Thrift determined that patients with BE had a lower likelihood of infection with *Hp* (OR = 0.37; 95% CI, 0.22–0.61) as was observed in many other studies. The BE group was then divided into two subgroups: BE without dysplasia and BE with dysplasia, and showed a reduced negative correlation (OR = 0.51; 95% CI, 0.30–0.86) and an increased negative correlation (OR = 0.10; 95% CI, 0.03–0. 33) when compared to population control, respectively [57]. Another case–control study with many more research objects further verifi ed this fin ding. When defining cases as BE with dysplasia or cancer, instead of simple BE, the negative correlation between *Hp* and the cases became stronger (OR = 0.31; 95% CI, 0.26–0.37 vs OR = 0.36; 95% CI, 0.34–0.38) [11]. However, a recent study in Azerbaijan, a high-prevalence area of *Hp* infection, directly compared BE with and without dysplasia, and found no significant difference in *Hp* infection between the two groups (OR = 0.42; 95% CI, 0.12–1.52; *P* > 0.05) [36]. Details of these studies are shown in Table 2.

**Table 2:** Characteristics of the four studies about the correlation between *Hp* and BE dysplasia

Authors	Years	Journal	*Hp* testing method	Biopsy location	BE	Cases	*Hp* +	Total	Controls	*Hp* +	Total
Aghayeva et al. [36]	2019	Dis Esophagus	H^*^, R^†^	Antrum	IM^‡^	BE with dysplasia	5	11	BE without dysplasia	48	72
Sonnenberg et al. [11]	2017	Aliment Pharmacol Ther	H	Stomach	IM	BE without dysplasia or cancer	1972	76,475	Endoscopy	20,683	284,552
						BE with dysplasia or cancer	138	6167	Endoscopy	20,683	284,552
Thrift et al. [57]	2012	Int J Cancer	S^§^		IM	BE	28	296	Population	73	390
						BE without dysplasia	25	208	Population	73	390
						BE with dysplasia	3	88	Population	73	390
Vieth et al. [65]	2000	Digestion	H	Antrum, Corpus	IM	BE	463	1054	NUD	378	712
						Barrett’s neoplasia (HGD|| or adenocarcinoma)	54	138	NUD	378	712

*: Histology, †: Rapid ureas e t est, ‡: Intestinal metaplasia, §: Serology, ||: High dysplasia

### Prevalen ce of CagA- positive *Hp* i n BE and controls

**Fig. 6:**
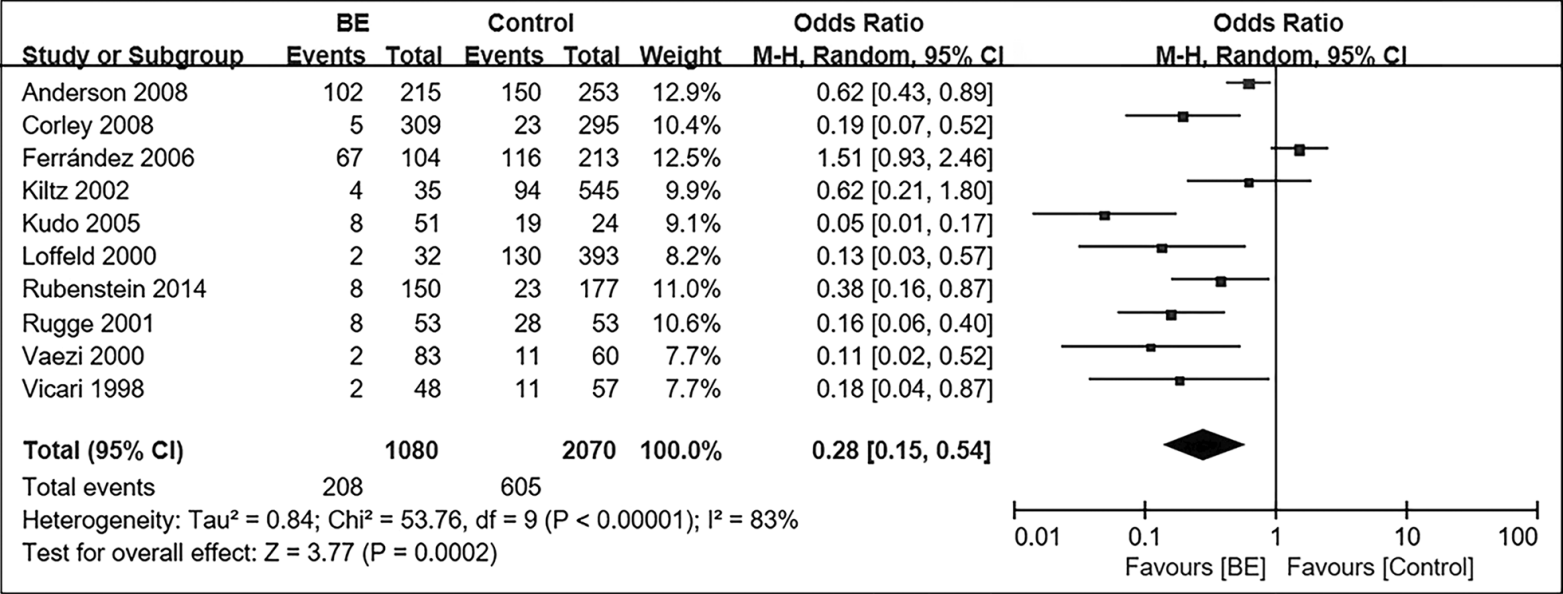
Forest plot of the correlation between the CagA-positive *Hp* strain and BE. The weights and heterogeneitie s of studies are also indicated. OR: Odds ratio, CI: 95% confidence interval

In the ten studies tha t examined patients with BE, the prevalence of the CagA-positive *H p* strain was significantly lower than that in controls (208/1080 [20.5%] vs 605/2070 [29.1%]) (OR = 0.28; 95% CI, 0.15–0.54, *P* = 0.0002; I^2^ = 83%) (Fig. 6) [12, 38, 45, 47, 54, 58, 59, 69, 71, 72]. In a case–control study in 2008, Corley confirmed that the inverse association between *Hp* and BE was stronger in subjects with the CagA-positive strain, weaker but still p resent in those with CagA-negative stra in [38]. Meanwhile, there were no substantial differences in the pattern of BE and the CagA-positive *Hp* stra in after adjustment for GERD symptom severity or GERD symptom frequency, which w as similar to Anderson’s conclusion [38, 69]. However, Anderson found a somewhat weaker pattern between the CagA-positive *Hp* strain and BE when analyzing for the CagA antig en only [69].

### Description of publication bias, heterogeneity, and sensitivity analysis

A visual inspection of the funnel plot was used to assess publication bias in the studies. There was no asymmetry in the funnel plots of the respective analyses and subgroup analyses. Considerable heterogeneity was noted in meta -analyses concerning the correlation between *Hp* prevalence and BE. Substantial heterogeneity was also noted when analyzing the relationship between *Hp* and lengths of BE, and th at between the CagA-positive *Hp* strain and BE. Through sensitivity analyses, we found that the significant heterogeneity could be attributed to factors other than a single study. We sometimes discovered decreased heterogeneity in the following subgroup meta-analyses. In the subgroup analysis of GERD, population and primary care people, the heterogeneity decreased considerably to 33% and 0%, respectively. This finding suggests that regarding subjects undergoing endoscopy as control might be the most potential sources of heterogeneity. There was also a significant decrease in heterogeneity when subgroup analysis was performed based on whether or not a match was made for sex and age. There were many factors closely related to *Hp* and BE, including sex, age, smoking, alcohol consumption, race, geographic location, definition of BE and control group, methods of *Hp* testing. It was hard to analyze and discuss each factor due to the limited number of publications and the heterogeneity of the description.

## Discussion 

In accordance with recent studies, our meta-analysis showed an inverse relationship between the prevalence of *Hp*, especially the CagA-positive *Hp* strain, with BE. The conclusions of most of the previous studies are consistent with those of the current study [14, 15, 77], in that *Hp* is a protective factor for BE. It is generally recognized that *Hp* causes corpus-predominant gastritis with decreased acid secretion, which is associated with a decreased risk of GERD and BE [78, 79]. Meanwhile, *Hp* infection reduces the chance of regurgitation by promoting gastric emptying and reducing the incidence of ob esity [79]. In subgroup analyses, *Hp* infection and BE were inversely related when compared with subjects undergoing endoscopy and normal control (population or primary care people), but not GERD control. Furthermore, the prevalence of *Hp* was not significantly different between patients with BE and those with GERD. Combined to previous studies, this protective effect of *Hp* is likely mediated by a decrease in prevalence of GERD in *Hp*-infected patients, since it disappears in patients with GERD [14]. However, there were no substantial differences in the relationship between BE and CagA-positive *Hp* strains after adjustment for GERD symptom seve rity or frequency [38, 71]. It suggested that CagA-positive *Hp* might reduce the risk of BE in some other ways.

Although *Hp* has been classified as a class 1 carcinogen, the majority of infected people had no symptoms associated with *Hp* infection actually [1]. Nowadays, the negative associations between *Hp* and asthma, allergies, GERD and inflammatory bowel disease are increasingly recognized [80]. The present study also revealed the protective effect of *Hp* on BE. Meanwhile, long-term use of proton pump inhibitors has been shown to increase the risk of gastric cancer after confounding factors, the HRs increased with cumulative duration, cumulative omeprazole equivalents and time since treatment initiation [81, 82]. Therefore, it would be important to explore new treatment options to alleviate BE symptoms and personalize *Hp* eradication.

The most likely protective mechanism of *Hp* to BE is the effect on gastric reflux by its influence on gastric acid secretion. Usually, antral-predominant gastritis is associated with increased acid secretion, whereas corpus-predominant gastritis, often accompanied by gastric atrophy, is associated with decreased acid secretion [83]. Ten previous studies only detected *Hp* infection with tissue from the antrum [13, 35, 36, 39, 44, 46–49, 55]; The meta-analysis of these arti c les showed *Hp* no protective impact to BE (OR = 0.80; 95% CI, 0.58–1.10; *P* = 0.17; I^2^ = 66%) although with decreased heterogeneity. In contrast, studies that defined *Hp* exclusively from esophageal biopsies tended to find a positive association between *Hp* and BE [18]. *Hp* directly damages the esophageal mucosa with bacterial products, increases the production of prostaglandin, sensitizes the afferent nerve, reduces the pressure of the lower esophageal sphincter, and increases acidity via Gastrin, an oncogenic growth factor that contributes to esophageal carcinogenesis [84–88]. Due to the lack o f classified discussion on the severity of gastric mucosal lesions after *Hp* infection in those included publications, our study is not able to prove the potential protective effect of *Hp* on BE might be explained by decreased acid secretion due to corpus-predominant gastritis. There are limited studies on the relationship between the duration, site, and severity of *Hp* infection and BE, and further disc ussions on classification are yet to be conducted.

In subgroup analyses based on different definitions of control and BE, we found that the inverse relationship disappeared when comparing BE with GERD control, and when BE was defined as a change other than IM. Conversely, the OR values of the other subgroups decreased to some extent. In particular, the prevalence of *Hp* infection in the normal control (population or primary care people) was much lower than that in patients with BE compared to the endoscopy subgroup. We also found that *Hp* was negatively correlated with LSBE, and that *Hp* infection could reduce BE dysplasia; however, there was no apparent correlation between *Hp* and SSBE. When it came to different detection methods for *Hp*, we found that the inverse relationship disappeared in the *Hp* infection history subgroup. Serological detection, treatment history, or infection history of *Hp* cannot reflect the current infection status of the study subjects, which will increase the uncertainty of information. In the present infected subgroup, our meta-analysis discovered a protective association between *Hp* and BE that was not present in the *Hp* infection history subgroup.

A few studies without obvious selection and information bias have reported a reduced risk of BE in people infected with *Hp* [18, 38, 53, 71]. The relationship between *Hp* infection and BE is controversial due to the considerable heterogeneity observed in most studies; indeed, significant heterogeneity was also noted in the current meta-analysis. A study by Fischbach et al. identified selection and information bias as potential sources of heterogene ity [71].

Subgroup analyses of the GERD and normal control (population or primary care people) showed a decrease of heterogeneity to 33% and 0%, respectively. The endoscopy subgroup might be one of the greatest sources of heterogeneity, since endoscopy might be associated with multiple gastrointestinal diseases. Applying subjects undergoing endoscopy, who were more likely to be colonized with *Hp* than the general population, as control, would lead to selection bias [38]; however, it also represents the most common and easiest control group. In the same way, blood donors cannot represent the population because they are likely to be healthier and younger [15]. Subject from the same geographical area as the BE patient would be the best choice of control.

A final, but no less important finding was that a significant decrease in overall heterogeneity was also observed when performing subgroup analyses based on whether or not a match was made for sex and age. Males and aging have been shown to be risk factors for *Hp* infection and BE, and in the current study, the protective effect of *Hp* infection wasn’t presented when matching both sex and/or age (OR = 0.72; 95% CI, 0.50–1.05; *P* = 0.09; I^2^ = 76%) [12, 13, 36, 38, 40, 44, 51, 60]. This result might be influenced by heterogeneity in definition of control group, definition of BE, *Hp* detection method, age, sex and so on. We collected information about whether or not the BE and control subjects were matched in sex, age, obesity, smoking, alcohol consumption, and race. However, it is unfortunate that, due to too many interfering factors, there were too few studies in single factor subgroups to perform additional subgroup analyses. The heterogeneity of existing studies is great, and a large number of rigorous and precise design studies are still needed to obtain more convincing conclusions.

## Conclusions 

In conclusion, the results showed a statistically significant inverse relationship between the prevalence of *Hp*, especially CagA-positive *Hp* strain, with BE. The prevalence of *Hp* was not significantly different between patients with BE and GERD controls, suggesting that this protective effect of *Hp* is probably mediated by a de crease in the prevalence of GERD. In addition, *Hp* was negatively correlated with LSBE, and *Hp* infection could reduce the BE dysplasia; however, there was no clear correlation between *Hp* and SSBE. In addition, th e inverse relationship between Hp and BE disappeared in the *Hp* infection history subgroup. The heterogeneity of existing studies is great. To understand the extent to which *Hp* reduces the risk of BE, further well-designed studies are needed. Researchers should pay attention to, but not only to, the definition of the control group, the definition of BE, status of *Hp* infection, sampling site, gastritis type, sex, age, obesity, smoking, alcohol, and race. 

## Data Availability

The datasets used and/or analyzed during the current study are available from the corresponding author on reasonable request.

## Abbreviations

*Hp*: *Helicobacter pylori*
BE: Barrett’s esophagus
OR: Odds ratio
CI: Confidence interval
GERD: Gastroesophageal reflux disease
LSBE: Long-segment BE
SSBE: Short-segment BE
USBE: Ultra-short-segment BE
EAC: Esophageal adenocarcinomas
CagA: Cytotoxin-associated gene A
NOS: Newcastle–Ottawa Scale
IM: Intestinal metaplasia
CM: Columnar metaplasia
S: Serology
R: Rapid urease test
U: Urea breath test
H: Histology
T: Treatment history
C: Culture
NUD: Non-ulcer dyspepsia
HGD: High grade dysplasia
SCJ: Squamous Columnar Junction
EGJ: Esophagogastric junction
